# Customizing virtual interpersonal skills training applications may not improve trainee performance

**DOI:** 10.1038/s41598-022-27154-2

**Published:** 2023-01-03

**Authors:** Gale M. Lucas, Johnathan Mell, Jill Boberg, Forrest Zenone, Ewart J. de Visser, Chad Tossell, Todd Seech

**Affiliations:** 1 Institute for Creative Technologies, University of Southern California, 12015 E Waterfront Dr., Los Angeles, CA 90094 USA; 2 School of Computer Science, University of Central Florida, Orlando, FL USA; 3 United States Military Academy at West Point, West Point, NY USA; 4 U.S. Air Force Academy, El Paso, CO USA; 5 Chief of Naval Air Training, Corpus Christi, TX USA

**Keywords:** Engineering, Human behaviour

## Abstract

While some theoretical perspectives imply that the context of a virtual training should be customized to match the intended context where those skills would ultimately be applied, others suggest this might not be necessary for learning. It is important to determine whether manipulating context matters for performance in training applications because customized virtual training systems made for specific use cases are more costly than generic “off-the-shelf” ones designed for a broader set of users. Accordingly, we report a study where military cadets use a virtual platform to practice their negotiation skills, and are randomly assigned to one of two virtual context conditions: military versus civilian. Out of 28 measures capturing performance in the negotiation, there was only one significant result: cadets in the civilian condition politely ask the agent to make an offer significantly more than those in the military condition. These results imply that—for this interpersonal skills application, and perhaps ones like it—virtual context may matter very little for performance during social skills training, and that commercial systems may yield real benefits to military scenarios with little-to-no modification.

## Introduction

Many virtual learning programs exist to teach social skills such as public speaking, negotiation, interviewing, and much more. Such programs are produced within academia—including public speaking trainings^1,2^ and negotiation trainings^3–7^—as well as produced by industry (for a review see^8^). For an example of the latter, the company Kognito creates a wide variety of virtual trainings for social skills around communication as well as awareness of mental health issues, LGBTQ issues, substance abuse and suicide prevention^9^. Often these companies “re-skin” the generic version of the training with cues that signal a specific affiliation (e.g., change agents to have proper outfits for the occupation, add company logo, etc.) to be able to offer a “customized” product.

Offering customized products implies that such superficial “reskinning” would have a positive, perhaps motivational, effect on users given that these systems made for specific use cases are more *costly* than generic “off-the-shelf” ones designed for a broader set of users. If there is no difference between these versions, organizations that purchase the training should seriously consider opting for the less expensive one. However, it remains an open empirical question whether these customized virtual trainings have such advantages over more generic ones. Accordingly, the claim that virtual context (or virtual environment) of social skills trainings should match the context in which the user is applying the training (e.g., the company or occupation in which they are employed) needs to be tested.

In this paper, we therefore manipulate the context of a virtual negotiation application. Specifically, military academy cadets who participate in the study were randomly assigned to complete a negotiation with an agent in military uniform over military-relevant items or with the same agent in civilian clothes over antiques. Out of 28 measures of performance in the negotiation, we find only one that differs significantly between the conditions. We therefore discuss how there may be little or no effect of virtual context in applications like this one.

We start by reviewing related work, first in terms of applied research and then more basic research relevant to our study. In the next section, we present the methodology used. Following that, we present the results, then discuss them in the subsequent section. We close the paper with three final sections: “Conclusions”, “Limitations” and “Future research” sections.

## Applied related research

While virtual learning programs have been developed within academia, they are also a common product produced by industry^8^. Kognito^9^, for example, creates a wide variety of virtual trainings for social skills. They have applications for communication around violence, bullying and death in the community; sexual misconduct, child abuse, neglect and trafficking; substance use; behavior management, emotional wellness, mental health; trauma, distress, suicidal ideation and moral distress; insular community youth; childhood nutrition; opioid use disorder and substance use; LGBTQ culture and gender affirmation; bloodborne pathogens; and chronic disease management.

Not only are there many different topics, but these trainings are used for extremely broad audiences—from teachers and students to government officials to heath care providers to family members of veterans. Such companies are often funded to create a “one-off” product in the sense that it is for a very specific target audience (e.g., one college campus). Indeed, many employees are quite familiar with the “customized” trainings given by their employers, as they often have mandatory trainings covering everything from leadership skills to sexual harassment prevention.

Often these companies make the 'one-off" product simply by “re-skinning” the generic version of the training with cues that signal a specific affiliation (e.g., change agents to have proper outfits for the occupation, add company logo, etc.). This approach is very common for the kind of social skill trainers described above, but it is not known whether this effort actually improves learners’ performance—although it is used as a selling point for a more expensive product.

The core argument for customization is that people learn better when the cues match their context; this is why, for example, Harvard’s negotiation training courses have negotiation cases where the terms and issues are relabeled to fit the context. If there is a negotiation training for the armed services, for instance, the standard negotiation case would be updated to include issues relevant to the military (e.g., a negotiation over “office supplies” could become “field rations”). However, we have not identified prior research that attempts to validate this approach by comparing a customized version to the non-customized version of the training. Rather, the commercial world creates this customization assuming that there is inherent value for learning and performance in doing so.

No applied work has scientifically considered this exact research question: does such context matter for virtual interpersonal skills training? The results of relevant research has been mixed. For example, while many companies have attested to the value of their realistic-looking agents, researchers found that users were more satisfied and happiest with their own performance in a simulation when they used very basic generic characters (i.e., one that moves around the environment in a static pose^10^;). Other developers in industry have explored the “ultimate” level of customization: personalizing the application for *each specific user*. For example, some trainings use “Mii” characters, where users can create an avatar that is a likeness of themselves. With a prior lack of research about whether customization made a difference in performance, it was believed that more detailed “reskinning” should increase user engagement. Data has emerged dispelling this belief; two sets of user studies both found that reskinning an avatar to look like a user does not have a significant effect on how users perform in virtual simulation^11,12^. Likewise, it is possible that user performance in customized versions of trainings, will not differ from that in the generic, non-customized version.

## Basic related research

### Theoretical underpinning for effects of context

The theoretical perspective of “situated cognition” offers insights about potential effects of the match between the virtual context and the context in which the user is taking the training. Generally, research on situated cognition has demonstrated that context changes perceptions and outcomes. This theoretical perspective posits that context in which an event occurs affects interpretation of the event, which in turn influences outcomes.

The theory has been applied to both social psychology and education. In social psychology, research has demonstrated that the environment or context can affect perceptions of others (e.g., for a review, see^13^). For example, research has examined various outcomes relevant to training from shoot/no shoot decisions (in military/law enforcement contexts) to language learning. For example, the type of neighborhood in which a user trains affects decisions to shoot during a law enforcement simulation^14^. Racial bias in shooting decisions against individuals with darker colored skin and more afro-centric features is exacerbated in more threatening neighborhoods (e.g., poorer) and reduced in less threatening neighborhoods (e.g., more affluent; see also^15^).

In education, the theory of situated cognition represents an approach to learning that emphasizes the importance of the social and cultural context in which learning occurs (for a review, see^16^). For example, when learning a language, the context in which the learner picks up the language will affect or “color” their understanding of the language (e.g., how the words are used in that kind of social interaction^17,18^;). Furthermore, people recall better when they are in the same context as when they learned the information (e.g.,^19,20^). There is also related evidence that, when we move to a new context where we have not been practicing a habit, that habit extinguishes (for a review, see^21^).

Overall, from this theoretical perspective of situated cognition, because context in which an event occurs affects interpretation of the event, the context has a large effect on responses in learning and in life. Moreover, because that interpretation—triggered by the context—would color how a skill is learned, the context of virtual social skills trainings should match the context where the skills are to be employed (e.g., same occupation, domain, setting, etc.) for best effects.

Like 'situated cognition", other theoretical approaches—like DIAMONDS and CAPTION—would also support the supposition that context matters. DIAMONDS^22^ and CAPTION^23^ are feature-based ways (i.e., taxonomies) to characterize psychologically-relevant aspects of the context to study (or “contextual variables”). While many taxonomies of person characteristics exist, there are fewer models of situations. Indeed, although DIAMONDS and CAPTION were developed fairly recently to outline and organize contextual variables, the need for these models reflects a much older debate in social psychology: the situation-person debate. “Situationist” researchers in this area became concerned with the fact that situations are much better predictors of behavior than the purely trait-based approaches to personality that did not take into account the situation. For example, Walter Mischel^24,25^ generated considerable debate in noting that observed correlations between measures of personality traits and related behaviors were fairly low, typically around 0.2 and rarely greater than 0.3.

This led Mischel and others to argue strongly for the relative superiority of contextual variables over individual differences in predicting human behavior. Given the importance of contextual variables, models like DIAMONDS and CAPTION were developed to list and organize them. They all presume though—like situated cognition—that the context would affect perception and thus learning outcomes; thus, it would be valuable to match the context for learning to the intended context where the learned skills would ultimately be applied.

### The effect of virtual environment on learning

This presumption—that the context for learning should match the intended context where skills will be applied—could apply to learning in virtual environments. However, work in that area has specifically considered, not the context, but rather the physical fidelity (likeness) of the virtual environment to the actual environment in which the learning is to be applied. Indeed, fidelity of virtual trainings—the degree to which the simulation visually replicates reality^26^—has received much attention. It was originally argued that virtual training with maximum fidelity would result in a level of transfer that is equivalent to real-world training because the two environments would be indistinguishable. Indeed, negative learning effects have been observed when there are problems with fidelity in simulations; for example, when buttons in vehicle or aviation simulators are in the wrong spot, a trainee might push the wrong button when operating the actual machine^27^.

Despite some evidence for such negative learning effects for specific cases like flight simulation, virtual training environments might not have to look exactly like the actual physical environments. Some have argued that an effective training environment doesn’t necessarily have to re-create exactly the real environment to be effective for training purposes^28,29^. Although virtual environments must be realistic enough to allow learning, they do not have to replicate the physical world in all respects. Indeed, TV and filmmakers create the feeling of being in the situation without needing a literal reenactment or re-creation. Supporting this latter claim, more recent research has found that the virtual context (in this case, recreation of the physical environment) matters very little as long as psychological fidelity is high (e.g., the virtual characters behave realistically).

In a recent review of the literature, Straus et al.^30^ found that, while some authors still argue theoretically for the importance of physical fidelity (i.e., the degree to which the virtual environment looks real), psychological fidelity (i.e., believability or “human-like” behavior of virtual actors) has been shown to be more important empirically. Even in the area of vehicle or aviation simulation, research has found no effect of physical fidelity on learning (e.g.,^31^). What’s more, other research suggests that people may sometimes learn better in virtual environments with lower physical fidelity (i.e.,^32,33^).

It is possible, though, that for some training objectives, low physical fidelity (or poor match to the intended context) may be sufficient, whereas for others, even small departures from reality could produce negative training. One main goal in virtual social skills trainers is for the learner to practice interpersonal skills. Then the question becomes, for this goal, what level of fidelity (or match to the intended context) is required for the social skills learned and practiced in the virtual training to transfer?

For such social skills training applications, too, there is better evidence for the importance of psychological fidelity (than physical fidelity), but even that evidence is mixed. Work has found that physical fidelity of the virtual actors (i.e., how well they are rendered) seems not to matter (e.g.,^34,35^) as long as they are represented as humans (e.g., given a face, even if it is low fidelity; e.g.,^36^). While some studies have shown an effect of psychological fidelity (e.g.,^37,38^), there are also some that show no impact of psychological fidelity on training (e.g.,^39–41^). Given this work, it is possible that the virtual context (recreation of the physical environment, appearance of avatars, etc.), and thus the extent to which that context is customized to the user’s context (e.g., organizational, social, etc.), might not matter much for the training outcomes.

### Present study

While theoretical perspectives like situated cognition and models like DIAMONDS and CAPTION would imply that the context of a virtual training should be customized to match the intended context where the skills would ultimately be applied, work on physical fidelity of the virtual context suggests this might not be necessary for learning. Specifically, we are interested in whether there are advantages in simply “re-skinning” generic trainings with cues that signal a specific affiliation, which amounts to fairly superficial contextual changes. It seems that work on physical fidelity of the virtual context may be more relevant here than these other theoretical perspectives.

Regardless, because these different perspectives would suggest opposing predictions (a difference vs. none) and the literature is unclear whether customizing context actually improves students’ performance, we test for a difference but do not specifically hypothesize that there will be one. Accordingly, we simply ask the research question of whether manipulating context matters for performance in a social skills training application.

In the present research, we explicitly test this question using a case that has important real-world implications: a virtual platform for members of the military to practice their negotiation skills. Leadership and teamwork are important goals to the United States Military (for example, see^42^); helping members of the military improve their negotiation skills directly works towards such goals. The military also generally distinguishes between environments: “home”, “Forward Operating Base”, and “on the front line”—and each of these environments carries with it different social norms, expectations, and perceptions. This may carry over to training in virtual environments such that recreating one of these locations could trigger such expectations^43^ and therefore affect the course of that particular virtual training (as suggested by^44^).

In these military training applications, the nature of the training environment is usually predetermined by the operational environment (i.e., where the skills will be deployed). The virtual environment would thus be customized to match the eventual physical locations for every mission. While this would be essential for mission planning activities, it might not be needed for training applications. Given the potential to expend a significant amount of effort and money customizing virtual environments for new physical locations, it would be important to determine for what kinds of training applications a customized virtual environment matters.

An important contextual distinction to military members is between being home and deployed and, even more generally, between on-duty (military) and off-duty (civilian) life. These two contexts differ wildly for warfighters; therefore, they serve as ideal comparison conditions for this case. While testing the effect of military context (e.g., military vs. civilian) seems simplistic, if these kinds of contextual variables are found not to matter, then it is highly unlikely that more nuanced factors (e.g., one village in Iraq vs. another) would have an effect. Moreover, the military versus civilian contextual variable is relatively straightforward to operationalize cleanly. The same virtual platform to practice negotiation can be used, with simple but observable differences between conditions to cue context.

Finally, and perhaps most importantly, this particular manipulation has special value for the case at hand: companies earn a great deal of money from customizing social skill trainings for various organizations (including the Armed Services). However, if off-the-shelf virtual trainings designed for civilians might work about as well for military members as customized versions do, then the cost–benefit tradeoff seems clear. Here, we consider the possibility that a generic negotiation training set in a civilian context may (or may not) lead to different performance in the negotiation practice for on-duty military members.

We therefore conducted a study in which military cadets all interacted with the same virtual platform to learn about and practice negotiation, and they were randomly assigned to either military or civilian context. Specifically, the appearance of the counterpart (virtual agent wearing uniform vs. civilian clothes) and the issues in the negotiation (negotiating over military supplies vs. antique collectables) are used to manipulate context; all other aspects of the platform are identical across conditions so as to have tight experimental control.

## Methods

The methods were reviewed and approved by the University of Southern California, the Army’s Human Research Protection Office, and the United States Air Force Academy’s human subjects protection board. All methods were performed in accordance with the IRBs’ guidelines and regulations. Additionally, the study protocol promised anonymity in two ways. First, it included not asking survey questions of participants; only their performance in the interpersonal skills training was tracked. Furthermore, procedures were used so that the cadets’ identifying information was not only never connected to the data, it was never seen by the researchers. Participants were automatically granted credit in the academy’s online research credit tracking system upon completion of the study. Participants also read an informed consent information sheet, and then clicked “agree to participate” in order to give informed consent.

### Sample

One hundred and two participants were recruited from the subject pool at the United States Air Force Academy. All cadets at this academy were eligible to participate; extra credit in their course(s) was offered as compensation for their participation. There is no demographic (gender, age, etc.) information available for these participants due to the authors’ and the academy’s intention to keep the study subjects completely anonymous. While the exact number of female participants in the sample is therefore unknown, the subject pool from which the sample was drawn is representative of the military academy at large, which has 28% female students.

### Variables

The independent variable was “virtual training context.” Specifically, cadets were randomly assigned to one of two conditions that differed only in terms of context: military versus civilian. Twenty eight dependent measures were taken to measure performance in the interpersonal skills training, specifically a training for negotiation skills that is described below. We outline all of these measures in “Appendix 1”, and analyze them below in the “Results” section.

### Instruments and materials

Cadets completed the study on their own personal computers. No survey questions were asked of participants.

**Figure 1:**
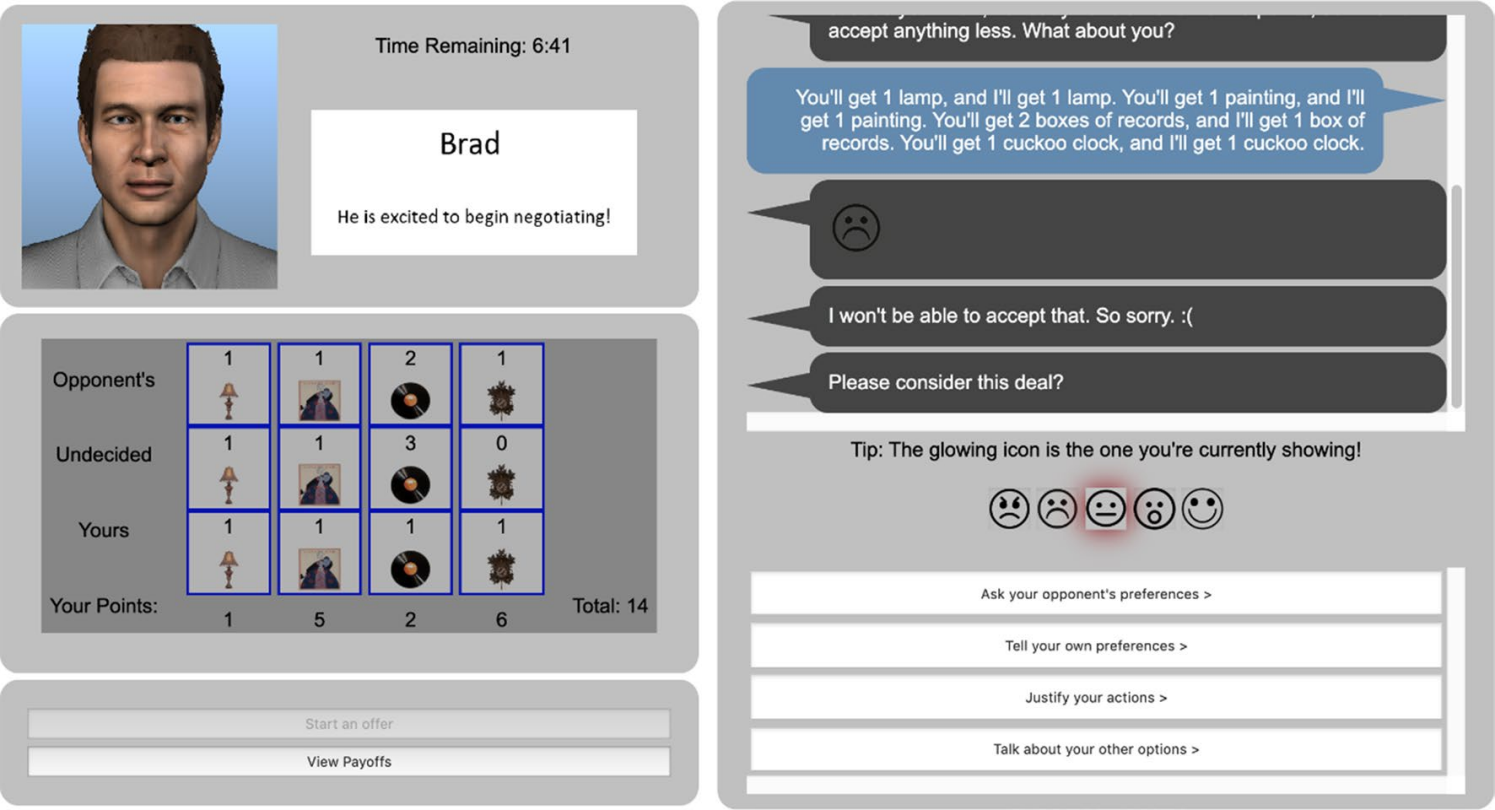
The interactive arbitration guide online.

In this study, all participants completed a training session with a virtual agent that acts (i.e., role-plays) as a negotiation partner. Specifically, the study used the Interactive Arbitration Guide Online (IAGO^45^; see Fig. 1). The IAGO platform provides a web-based negotiating interface between an artificial agent and a human player, and allows complex agents to be designed to act within that interface.

Specifically, IAGO implements the “multi-issue bargaining task,” one of the standard tasks used to practice negotiation in classroom settings^46–48^, and now in virtual settings as well (e.g.,^4–6^). In this task, a number of items are assigned to be split between each of the two negotiating parties—the human user and the virtual human component. Each side is aware of how much the items are worth to them, but are unaware of how much the same items are worth to their opponent. Furthermore, each side is given a value called the “Best Alternative To Negotiated Agreement” or BATNA, which represents the number of points they would receive if no agreement is reached within the allotted time.

Each party must then communicate using a set of pre-written natural language phrases, emotional display buttons, preference questions and statements. Negotiators may also use the “game board” (see Fig. 1) to send proposed offers in which they split the items, and may respond positively or negatively to received offers. The negotiation ends when all the items are split (leaving no items up for grabs) or when the 7 min timer expires.

IAGO allows this task to be performed on a web browser, and is easily distributed to online subject pools, such as Amazon’s Mechanical Turk (MTurk), universities, or academies. Furthermore, detailed logs and data regarding human and agent performance is collated. The 28 dependent measures mentioned above were specifically logged by the IAGO system, reflecting the decisions of the user and their outcomes. All extracted measures that had at least one user select the option were analyzed (variables where means for both groups were zero are excluded).

Participants interacted with one of the IAGO agents (from^45^) that are provided with the platform itself. The agent is fundamentally dynamic and adaptive in its design. For example, it has an internalized conception of “fairness” based on its internal mental model of the opponent’s preferences, which is updated as users make additional statements about their preferences. This agent looks for a moderately-sized positive margin over the user. However, adverse events (such as offer rejections) will reduce this margin over time (details can be found in^45^). Based on this policy, the agent decides if it wants to accept, reject, or ignore the offer, as well as what it should say (e.g. “Yes, that offer sounds good to me!” or show an emoji; see^45^, for how these policies are crafted).

Importantly, this same agent with the identical policy for behavior during the negotiation was used in both experimental conditions (see “Experimental manipulation” section below). This means that while the negotiation was fully interactive (and therefore varied based on user actions), there were no systemic differences between the behavior of the agent—indeed the agent was fully deterministic, and the same set of actions would lead to the same behavior across conditions.

### Procedures

Upon signing up for the study via the academy’s online research credit tracking system, the students were provided with a link to the study. Participants first read the informed consent information sheet, and then clicked “agree to participate” in order to give informed consent.

Upon reading and agreeing to the information sheet, indicating their consent without collecting identifying information, all participants were given a tutorial of IAGO, which not only included how to use the platform to negotiate with the virtual agent, but also best practices on how to negotiate (e.g., make a high initial offer; see^6^). While the military rank of the IAGO agent was not specified in the military condition, the participants were all told that this agent was to be their “negotiation partner” for the duration of the study.

Participants then interacted the IAGO agent above^45^. Upon completion of the negotiation, participants were then thanked and granted credit for their participation in the academy’s online research credit tracking system.

### Experimental manipulation

**Figure 2:**
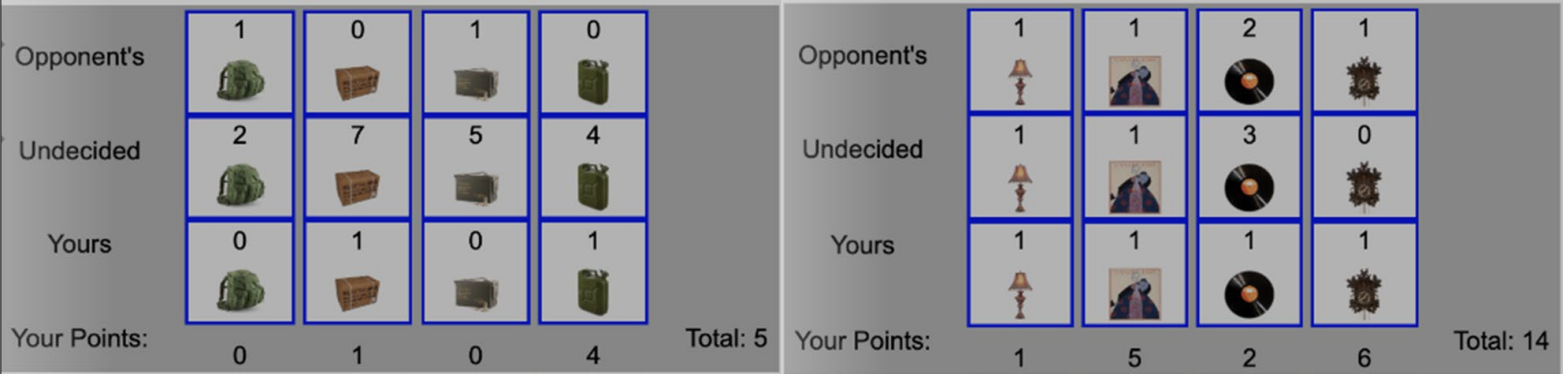
Items negotiated over in the military versus civilian conditions.

**Figure 3:**
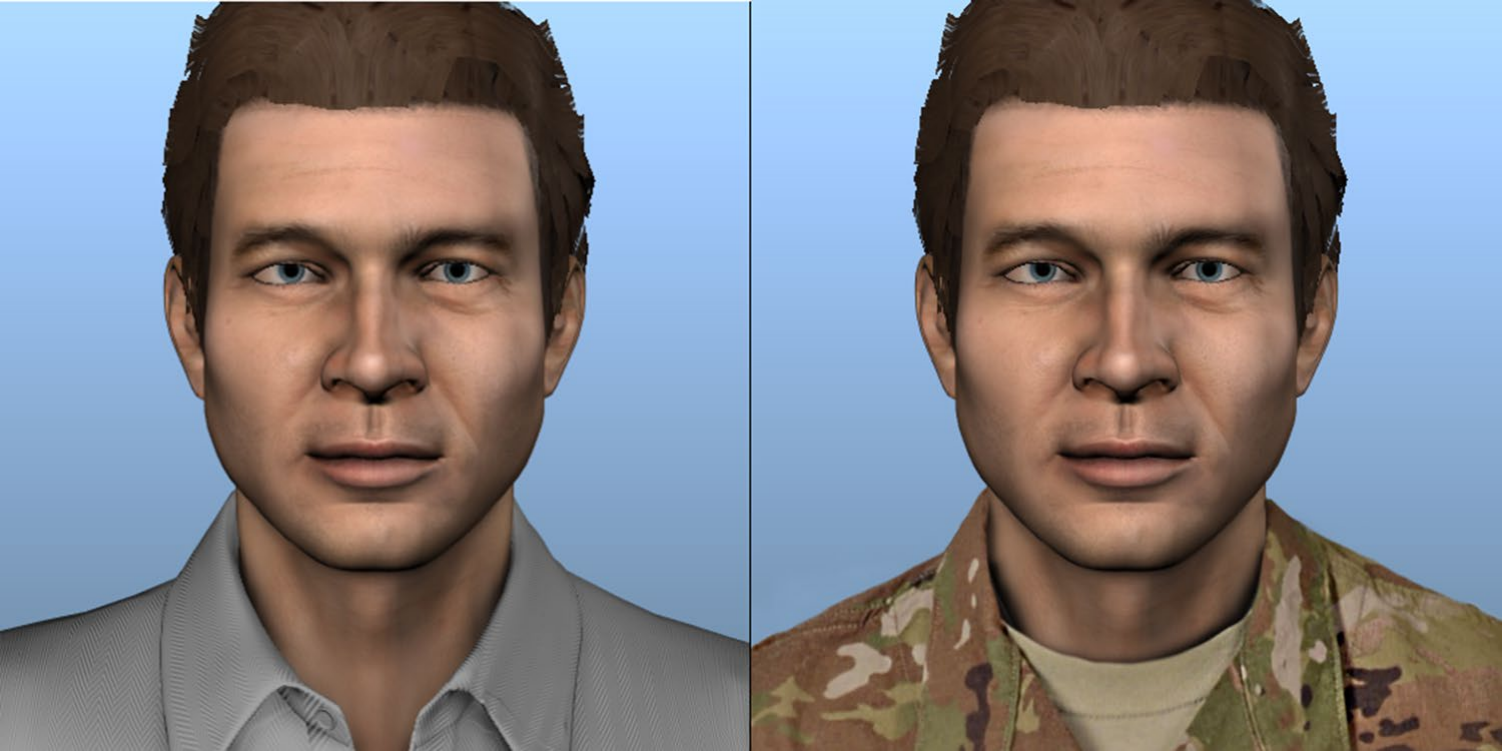
The agent in the civilian versus military conditions.

Recall that, in the study, cadets were randomly assigned to one of two conditions that differed only in terms of context: military versus civilian. As displayed in Fig. 2, in the civilian condition, the agent and the user negotiated over lamps, paintings, records, and cuckoo clocks, whereas in the military condition, they negotiated over equipment sets, MRE cases, ammunition boxes, and fuel cans. These military items were chosen due to the roughly equivalent value to each other (e.g., compared to some much higher value items, such as missiles). Furthermore, the point values chosen for these items were pilot tested with the help of cadets for how realistic they would be to their peers. Final point values used in the study reflect agreement between pilot testers on appropriate relative point values. Additionally, as shown in Fig. 3, the agent wore a polo shirt in the civilian condition, and wore fatigues in the military condition. In both conditions, distribution of points for items (for agent and for user) were equivalent, the number available of each (respective) item, and the behavior of the agent was identical (as described above); only the context of the negotiation (appearance of the agent, names of the items) differed between the experimental conditions.

## Results

For each measure extracted by IAGO about the user’s behavior and outcomes in the negotiation, an independent samples t-test was conducted. Means and standard deviations for each condition are provided in Table 1, as well as the t-values and *p* values for comparing the two experimental conditions. As can be seen in the table, only one effect reached statistical significance: the number of times the user asks for an offer using a positive tone. Cadets in the civilian condition ask the agent to make an offer significantly more using a polite tone (e.g., “Would you please make an offer?”) than those in the military condition (t(100) = 2.69, *p* = 0.008). Also, there was no statistical difference between conditions in the number of participants who failed to reach agreement (χ2 (1) = 0.65, *p* = 0.42).

**Table 1:** Means and standard deviations for experimental conditions with T Value for the comparison.

	Means and SDs		Test statistics	
	Civilian	Military	t	*p*
Total points earned (from items claimed)	27.48 (5.09)	28.44 (6.54)	− 0.72	0.47
Time spent in the negotiation	317.78 (109.90)	334.58 (96.54)	− 0.82	0.42
Number of offers made	3.94 (2.52)	4.38 (4.15)	− 0.64	0.52
Number of times neutral emoji used	0.44 (0.84)	0.46 (0.71)	− 0.09	0.93
Number of times happy emoji used	1.07 (1.43)	1.31 (1.32)	− 0.87	0.39
Number of times angry emoji used	0.70 (1.73)	0.88 (3.22)	− 0.34	0.74
Number of times surprised emoji used	0.41 (0.69)	0.40 (0.64)	0.09	0.93
Number of times sad emoji used	0.30 (0.60)	0.48 (0.68)	− 1.44	0.15
Total number of utterances made (all below; no emoji, offer)	7.43 (4.56)	7.31 (3.94)	0.13	0.89
Total number of utterances with preferences (incl. question)	3.46 (2.17)	3.69 (2.64)	− 0.47	0.64
Number of statements made about preferences	1.85 (1.56)	2.02 (2.09)	− 0.47	0.64
Number of questions asked about preferences	1.61 (1.50)	1.67 (1.43)	− 0.19	0.85
Number of requests for information	0.11 (0.46)	0.06 (0.25)	0.65	0.52
Number of false statements about preferences (pref. lies)	0.28 (0.76)	0.27 (0.68)	0.05	0.96
Number of false statements about BATNA (BATNA lies)	0.13 (0.39)	0.08 (0.28)	0.68	0.50
Number of true statements made about own BATNA	0.26 (0.52)	0.21 (0.41)	0.54	0.59
Number of questions asked about agent’s BATNA	0.26 (0.48)	0.35 (0.53)	− 0.95	0.34
Number of threats made that are phrased negatively	0.06 (0.23)	0.04 (0.20)	0.32	0.75
Number of threats made that are phrased positively	0.04 (0.19)	0.00 (0.00)	1.35	0.18
Number of statements indicating confusion	0.02 (0.14)	0.02 (0.14)	− 0.08	0.93
Number of times user requests a favor	0.31 (0.61)	0.38 (0.57)	− 0.51	0.61
Number of times user says they’re doing them a favor	0.17 (0.47)	0.25 (0.48)	− 0.89	0.38
Number of generic negative statements made	0.19 (0.44)	0.13 (0.39)	0.73	0.47
Number of generic positive statements made	0.50 (0.84)	0.31 (0.93)	1.07	0.29
Number of times user accepts an offer (incl. partial offers)	0.72 (0.98)	0.63 (1.08)	0.48	0.64
Number of times user rejects an offer	0.94 (1.24)	1.15 (1.26)	− 0.82	0.42
Number of times user requests an offer phrased negatively	0.02 (0.14)	0.02 (0.14)	− 0.08	0.93
Number of times user requests an offer phrased positively	**0.22 (0.57)**	**0.00 (0.00)**	**2.69**	**0.008**

Significant values are in bold.

Although independent samples t-tests are robust to violations of assumptions with large enough sample sizes (such as ours), we tested for such violations to determine if they were contributing to the observed results. We first considered the equality of variance assumption. The Levene’s test for Equality of Variances shows that variance in the number of times the user asks for an offer using a positive tone is significantly lower in the military condition than the civilian condition (F = 35.12, *p* < 0.001). However, the inequality of variances does not account for the observed difference in the number of times the user asks for an offer using a positive tone. Indeed, running a t-test with a Satterthwaite approximation for the degrees of freedom does not change the result (*t*(53) = 2.86, *p* = 0.006, down from 0.008 with equal variances assumed). A Levene’s test on the number of threats made that are phrased positively was also significant (F = 7.83, *p* = 0.006). Again, a t-test with a Satterthwaite approximation for the degrees of freedom did not reveal a different result (*t*(53) = 1.43, *p* = 0.16, down from 0.18 with equal variances assumed). The Levene’s test scores did not reach significance for any other measures (Fs < 2.80, *p*s > 0.10).

Considering the normality assumption, none of the distributions were normal (Shapiro–Wilk test scores > 0.12, *p*s < 0.001), however, small deviations from normality can produce statistically significant *p* values when the sample sizes are large, so we examined normality using q–q plots. Non-normal distributions observed using the q–q plots were driven by significant outliers (identified using Tukey’s method).

Removing these outliers resulted, on average, in similar levels of statistical significance. Removing outliers resulted in only one effect approaching significance: in the civilian condition, cadets tended to use more generic positive statements (M = 0.38, SD = 0.60) than in the military condition (M = 0.19, SD = 0.40; *t*(97) = 1.87, *p* = 0.07, down from 0.29 without outliers removed).

Although differences between conditions in emoji use did not become significant (*p*s > 0.20), extreme use of emojis by a few participants did contribute *somewhat* to the null results above; once outlier scores were removed, the *p* values did drop. However, the emoji measure that was previously closest to significance (sad emoji use, at *p* = 0.15) raised (to *p* = 0.28) upon removal of outlier scores. Outside of emojis, the significance level dropped only for the number of favor requests (from *p* = 0.61 to 0.29). For all of the 17 other measures for which significant outliers were found, *p* values increased upon removal of these outlier scores; in particular, the t-test for number of times the user asks for an offer using a positive tone was no longer significant (*p*s > 0.62). Accordingly, it seems that outliers usually made the effects *more* significant. Thus, outliers generally do not explain the null effects reported above.

## Discussion

Across these measures, a clear pattern emerged: other than the one significant result, performance in the negotiation was unaffected by context. Specifically, cadets in the civilian condition ask the agent to make an offer significantly more using a polite tone (e.g., “Would you please make an offer?”) than those in the military condition (t(100) = 2.69, *p* = 0.008).

This single effect is highly significant so, although it could of course be due to chance, there is a low likelihood (8 out of 1000) that it is a type I error or false positive result. As to an explanation, it is possible that asking (rather than being “told”) is more expected in civilian settings than military ones due to the strict vertical hierarchy of the military. Indeed, while some cadets asked the agent politely to make an offer in the civilian condition, not a single cadet in the military condition asked for an offer in such a way from the agent. This effect did not occur because those in the military condition asked the agent to make an offer *using a negative tone* instead; there was no significant difference for that variable (t(100) = − 0.08, *p* = 0.93), and very few participants chose to ask for offers using a negative tone in either condition.

Furthermore, while unequal variances were observed for the number of times the user asks for an offer using a positive tone, correcting for unequal variances did not change the result. In contrast, removing outliers resulted in the effect no longer being significant, casting doubt on even this one significant finding.

All the other results were not significant or even marginally significant. Therefore, our results suggest that superficial differences in the simulation to signal a different context (e.g., civilian vs. military) may not matter much for performance in a social skills training application like IAGO. Because a manipulation of military context (e.g., military vs. civilian) seems not to matter for learning such skills in IAGO, then it is highly unlikely that more nuanced factors (e.g., one village in Iraq vs. another) would have any effect on performance in this platform. Given the potential to expend a significant amount of effort and money customizing virtual trainings for new contexts, this work begins to identify which social skills training applications are sensitive (or in this case not sensitive) to the virtual training context.

From these results, one could conclude that using off-the-shelf, “generic” (in this case, civilian context) trainings could save money, time, and expertise when compared to customized trainings and not compromise learning—at least for negotiation skills tested by IAGO. Specifically, developers (like those mentioned in the introduction) often already have a civilian version of the interpersonal skills training on offer, and to fit the training for military members, they will (at cost) create a version of the same training with the agents dressed as military members, and change the scenario to be relevant to the military. However, our results seem to imply that, when such companies “re-skin” the generic version of the training with cues that signal a specific affiliation (e.g., change agents to have proper outfits for the occupation, add company logo, etc.), it could have little or no impact on learners’ performance in applications like IAGO.

Beyond this practically-minded outcome, the present work also contributes to research on both human–computer interaction and psychology. First, this work adds to the literature on physical fidelity by reinforcing the idea that, for many training outcomes, the virtual training may not need to perfectly recreate the situation in which the skill will be applied. It also implies that at least some contextual variables (i.e., military vs. civilian) may not be essential for psychological fidelity either.

Moreover, there is relatively little work on the effect of the virtual context, specifically, on training outcomes, and this research therefore contributes novel findings to this relatively new area exploring the effect of virtual environment on learning. In contrast, the present findings do not provide support for the theoretical perspective of situated cognition where context affects learning outcomes. It is possible that this perspective is more relevant to real-world than virtual conditions for learning.

## Conclusions

Using the case of a virtual platform to practice negotiation skills, our study with military cadets suggests that virtual context (specifically, military vs. civilian in this case) may have little impact on performance in certain virtual training applications. This work calls into question whether *all* customized virtual trainings made for specific sets of users have advantages over more generic ones that are for a broader set of users. Instead of customizing the virtual context, it is possible that, at least for some applications, off-the-shelf virtual social skills trainings might produce similar learning outcomes as customized ones, and resources could be better allocated elsewhere.

## Limitations

Although the study showed 27 out of 28 outcomes were not significantly different between civilian and military contexts, it is still possible that there is an effect of context on user performance or behavior. We could have failed to find it due to chance. The authors view this as unlikely, as we had a sufficient sample size to detect a moderate effect. To the extent that the effect is smaller, we would have had a greater chance of failure to detect the effect with our given sample size. Power analyses based on the current sample size show that, if there is an effect, it is most likely quite small (d ~ 0.1–0.2). The practical significance of such a small effect would be limited. Even if such an effect does exist, it may not be large enough to warrant the expense of customizing virtual social skill trainings just to reap (such small) benefits on performance and behavior.

Additionally, the location where subjects used the computer simulation could impact their responses. However, that context is static across conditions: all participants completed the study during the mandated “work from home” orders at this academy. Therefore, participant physical location or context was invariant across conditions and could not have contributed to any differences across conditions. However, future work could consider if the combination of virtual and physical environments has an interactive effect.

Finally, while the cadets are indoctrinated into military culture through various experiences (e.g., basic training) before they even come to campus, cadets would be less acculturated in general than service members who have already gotten out of the academy. Future work should therefore examine if there is a moderating effect of acculturation in order to identify (or rule out) that more acculturated members of the military show more effect of context.

Likewise, another limitation of the current design is that there is only a military sample, rather than a civilian and military sample. This is a limitation because military personnel have the experience of being in military and civilian contexts, whereas most civilians only have experience in civilian (and not military) contexts. Therefore the current work does not allow for testing whether experience in the context moderates the effect of context, and future work should do so.

However, the current design was specifically chosen to address the singular question as to whether there is a benefit of simply “re-skinning” a generic version of interpersonal skills training to match a specific population (i.e., “re-skin” for military context for population of warfighters). The generic version would already be appropriate for civilians, so would not require re-skinning. Because our motivation is to consider the benefit to users when designers try to sell “re-skinned” versions of the training (given the higher cost), we consider only military personnel here as they are a population that could (and has) been targeted for such “re-skinned” training.

It is also a limitation that “re-skinning” in this way is a weak manipulation of context. While it is indeed true that stronger manipulations of context could have an effect, the current design tests whether there is a benefit of only “re-skinning” a generic version of interpersonal skills training to match a specific population. Future work should consider other, stronger manipulations of context to further contribute to the literature on the effect of virtual context; however, this work does suggest that more superficial and limited manipulations of context, such as “re-skinning”, might not impact learning outcomes even if stronger manipulations do.

Finally, even within social skills trainings, there are variations in the nature of the skills being trained. For negotiation, lots of social skills are relevant. Skills such as empathy or emotional regulation are “soft skills” when compared to skills like identifying opportunities to make trade-offs for win–win deals or discerning the other person’s BATNA. IAGO’s measures of performance tap these “harder” social skills within negotiation. Other trainings like Harvard’s negotiation courses also focus on these latter skills when teaching negotiation. Accordingly, their “selling point” of relabeling their standard negotiation cases to match the occupation of the trainees might not be superior to just using the non-customized standard negotiation case itself.

On the other hand, it is possible that context affects learning of “soft skills” even though we found no effect for these harder skills used in negotiation. Indeed, the military context may evoke very different notions or perceptions of such soft skills than a civilian one, and may have seen a difference. Our findings might not, then, generalize to soft skills for negotiation, and therefore future research should consider both sets of skills relevant for negotiation.

## Future work

While negotiation training appears not to be sensitive to context effects (or at least the effect would be very small), there might be significant effects of the virtual environment for other kinds of interpersonal training. Furthermore, non-social skills (from mathematics to wayfinding) are beyond the scope of the current work, and should be considered in future work on learning in those disciplines.

Additionally, it is possible that other tasks would show a larger, and thus perhaps statistically significant, effect of context, and even could be sensitive to more fine-grained differences in context or environment. It also could be that, for other social skills, learning may even be affected by context (e.g., matching to social setting) but not environment (physical location recreated in virtual training). Research is needed to investigate these possibilities by examining cases other than the one considered here.

By discovering for which kinds of skills virtual context matters (vs. does not matter), future research could be informative in guiding design decisions when developing virtual training applications. For example, imagine that results demonstrate that virtual context has an impact when the setting is essential to the task (i.e., giving clear directions from one location to another), but has no impact when the environment is not essential to the task (i.e., negotiation). This would suggest “how essential the context is to the task” might be a moderating factor.

If future research can find such moderators, it could have important impacts for generalizability: designers might choose, based on this result, not to customize their virtual environment even though their specific task was not tested empirically because they applied what was learned by the moderation studies (e.g., “how essential the context is to the task”). Generalizing based on such observations is far more efficient than testing every type of context, task, etc. (albeit there are also additional sources of potential error compared to testing each context, task, etc.). Accordingly, basic research like the present study can serve an important role in guiding design choices for applied settings (like virtual training for the Armed Services).

Another conceivable limitation of the current work lies in the possibility that, if cadets believed their counterpart were human, then the distinction between military versus civilian would have mattered more. Perhaps negotiating with another service member would be experienced differently than a civilian—but a virtual agent cannot be in the military, so context had no effect in this case.

Prominent theory—and related evidence—in human–computer interaction would argue otherwise, however. Making a case for psychological fidelity, the Media Equation posits that people will respond fundamentally to media (e.g., fictional characters, cartoon depictions, virtual humans) as they would to humans as long as it seems to behave realistically. For example, when asked to rate the performance of an advice-giving agent, users tried to be as polite as with humans^49^, yet, other virtual objects (without human-like features) are not afforded such considerations^50^. Subsequent research further supports the idea that we treat virtual agents in learning applications like we would humans^51–53^, and some research finds that we apply social labels (like military vs. civilian) to agents^54–57^. So, while virtual agents cannot be in the military (yet), we appear to have no problem with them acting as if they are^58^, and treat them accordingly.

Even so, if one were to conduct an empirical test to determine whether the current study found little effect of context because cadets negotiated with an agent (rather than a human), they could do so by manipulating “perceived agency”—whether the user thinks their counterpart is a real person or artificial intelligence^59^. Perceived agency is about who or what users believe is “behind the curtain”: avatars are operated by human users, whereas agents are controlled by artificial intelligence of some kind^60^. Both agents and avatars are depicted as media, whether it be cartoon depictions or realistic-looking virtual humans; in fact, an agent and an avatar could look identical. Therefore, future work could replicate and extend the current work by adding a manipulation of perceived agency where users are led to believe the virtual agent from the current study is operated by either a human or computer.

However, as long as psychological fidelity is intact^37^, how much this framing (agent vs. avatar) matters is up to question. Indeed, when psychological fidelity was explicitly manipulated for both an agent and avatar, people treated the more realistic agent like a human regardless of whether they believed it was an agent or an avatar (Ref.^38^; see also^51–53^). Again, psychological fidelity seems to reign supreme over other factors. In sum, then, while it would be interesting to add perceived agency as a factor, this prior work suggests that it may not matter. Therefore, such research might find—like we do here—that virtual context has little effect on performance in our specific virtual learning environment.

## Supplementary Information

Supplementary Information.

## Data Availability

The datasets generated during and/or analyzed during the current study are available from the corresponding author on reasonable request.

## References

[1] Chollet, M., Wörtwein, T., Morency, L. P., Shapiro, A. & Scherer, S. Exploring feedback strategies to improve public speaking: an interactive virtual audience framework. In *Proceedings of the 2015 ACM International Joint Conference on Pervasive and Ubiquitous Computing*, 1143–1154 (2015).

[2] Kang N, Brinkman WP, van Riemsdijk MB, Neerincx M (2016). The design of virtual audiences: Noticeable and recognizable behavioral styles. Comput. Hum. Behav..

[3] Kim JM, Hill RW, Durlach PJ, Lane HC, Forbell E, Core M, Marsella S, Pynadath D, Hart J (2009). BiLAT: A game-based environment for practicing negotiation in a cultural context. Int. J. Artif. Intell. Educ..

[4] Johnson, E., Lucas, G. M., Kim, P. H. & Gratch, J. Intelligent tutoring system for negotiation skills training. In *Proceedings of the International Conference on Artificial Intelligence in Education*, 122–127 (2019).

[5] Johnson, E., Roediger, S., Lucas, G. M. & Gratch, J. Assessing common errors students make when negotiating. In *Proceedings of the 19th International Conference on Intelligent Virtual Agents*, 30–37 (2019).

[6] Monahan, S., Johnson, E., Lucas, G. M., Finch, J. & Gratch, J. Autonomous agent that provides automated feedback improves negotiation skills. In *Proceedings of the International Conference on Artificial Intelligence in Education*, 225–229 (2018).

[7] Robb A, White C, Cordar A, Wendling A, Lampotang S, Lok B (2015). A comparison of speaking up behavior during conflict with real and virtual humans. Comput. Hum. Behav..

[8] Vaughan N, Gabrys B, Dubey VN (2016). An overview of self-adaptive technologies within virtual reality training. Comput. Sci. Rev..

[9] Coleman D, Black N, Ng J, Blumenthal E (2019). Kognito's avatar-based suicide prevention training for college students: Results of a randomized controlled trial and a naturalistic evaluation. Suicide Life-Threat. Behav..

[10] Normoyle, A. & Jorg, S. Trade-offs between responsiveness and naturalness for player characters. In *Motion in Games*, 61–70 (2014).

[11] Lucas, G. M., Szablowski, E., Gratch, J., Feng, A., Huang, T., Boberg, J. & Shapiro, A. The effect of operating a virtual doppelganger in a 3D simulation. In *Proceedings of the 2016 ACM SIGGRAPH Motion in Games Conference*, 167–174 (2016).

[12] Wauck H, Lucas GM, Shapiro A, Feng A, Boberg J, Gratch J (2018). Avatar self-similarity, performance, and subjective experience in a search and rescue game. CHI.

[13] Barrett LF, Mesquita B, Gendron M (2011). Context in emotion perception. Curr. Dir. Psychol. Sci..

[14] Kahn KB, Davies PG (2017). What influences shooter bias? The effects of suspect race, neighborhood, and clothing on decisions to shoot. J. Soc. Issues.

[15] Lee H, Jang H, Yun I, Lim H, Tushaus DW (2010). An examination of police use of force utilizing police training and neighborhood contextual factors: A multilevel analysis. Polic. Int. J. Police Strateg. Manag..

[16] Anderson JR, Reder LM, Simon HA (1996). Situated learning and education. Educ. Res..

[17] Brown JS, Collins A, Duguid P (1989). Situated cognition and the culture of learning. Educ. Res..

[18] Miller GA, Gildea PM (1987). How children learn words. Sci. Am..

[19] Byrne JH, Kandel ER (1996). Presynaptic facilitation revisited: State and time dependence. J. Neurosci..

[20] Jansen LC, Harris K, Anderson DC (1971). Retention following a change in ambient contextual stimuli for six age groups. Dev. Psychol..

[21] Wood W, Neal DT (2009). The habitual consumer. J. Consum. Psychol..

[22] Rauthmann JF, Gallardo-Pujol D, Guillaume EM, Todd E, Nave CS, Sherman RA, Ziegler M, Jones AB, Funder DC (2014). The situational eight DIAMONDS: A taxonomy of major dimensions of situation characteristics. J. Personal. Soc. Psychol..

[23] Parrigon S, Woo SE, Tay L, Wang T (2017). CAPTION-ing the situation: A lexically-derived taxonomy of psychological situation characteristics. J. Personal. Soc. Psychol..

[24] Mischel, W. Consistency and specificity in behavior. In *Personality and assessment*, 13–39 (1968).

[25] Mischel W, Peake PK (1982). Beyond déjà vu in the search for cross-situational consistency. Psychol. Rev..

[26] Hays RT (1980). Simulator Fidelity: A Concept Paper (No. ARI-TR-490).

[27] Waller D, Hunt E, Knapp D (1998). The transfer of spatial knowledge in virtual environment training. Presence Teleoper. Virtual Environ..

[28] Swartout, W. R. & Lindheim, R. Does simulation need a reality check? In *Proceedings of the Summer Computer Simulation Conference*, 917–921 (2003).

[29] Swartout, W. R. & Lindheim, R. Does simulation need a reality check?. In *Proceedings of the Workshop on the Scientific Exploration of Simulation Phenomena*, 61–65 (2003b).

[30] Straus SG, Lewis MW, Connor K, Eden R, Boyer ME, Marler T, Carson CM, Grimm GE, Smigowski H (2019). Collective Simulation-Based Training in the US Army.

[31] Estock JL, Alexander AL, Stelzer EM, Baughman K (2007). Impact of visual scene field of view on F-16 pilot performance. Proc. Hum. Factors Ergon. Soc. Annu. Meet..

[32] Badariah, S. & Mania, K. The effect of visual fidelity on transfer of training and awareness states. In *Proceedings of the 2nd Symposium on Applied Perception in Graphics and Visualization*, 173–173 (2005).

[33] Mania K, Troscianko T, Hawkes R, Chalmers A (2003). Fidelity metrics for virtual environment simulations based on spatial memory awareness states. Presence Teleoper. Virtual Environ..

[34] Volonte, M., Duchowski, A. T. & Babu, S. V. Effects of a virtual human appearance fidelity continuum on visual attention in virtual reality. In *Proceedings of the 19th ACM International Conference on Intelligent Virtual Agents*, 141–147 (2019).

[35] Wang, Y., Khooshabeh, P. & Gratch, J. Looking real and making mistakes. In *Proceedings of the 13th International Conference on Intelligent Virtual Agents*, 339–348 (2013).

[36] de Melo, C., Carnevale, P. J. & Gratch, J. Agent or avatar? Using virtual confederates in conflict management research. In *Proceedings of the Annual Meeting of the Academy of Management*, 1–30 (2013).

[37] Sheehan PW, Statham D, Jamieson GA (1991). Pseudomemory effects and their relationship to level of susceptability to hypnosis and state instruction. J. Personal. Soc. Psychol..

[38] Von der Pütten AM, Krämer NC, Gratch J, Kang S-H (2010). “It doesn’t matter what you are!: ” Explaining social effects of agents and avatars. Comput. Hum. Behav..

[39] Blascovich, J. A theoretical model of social influence for increasing the utility of collaborative virtual environments. In *Proceedings of the 4th International Conference on Collaborative Virtual Environments*, 25–30 (2002).

[40] Bailenson JN, Yee N, Merget D, Schroeder R (2006). The effect of behavioral realism and form realism of real-time avatar faces on verbal disclosure, nonverbal disclosure, emotion recognition, and copresence in dyadic interaction. Presence Teleoper. Virtual Environ..

[41] Heider F, Simmel M (1944). An experimental study of apparent behavior. Am. J. Psychol..

[42] US Army. *Army Warfighting Challenges*. http://arcic-sem.azurewebsites.us/initiatives/armywarfightingchallenges (2018).

[43] Rizzo AA, Graap K, Perlman K, McLAY RN, Rothbaum BO, Reger G, Pair J (2008). Virtual Iraq: Initial results from a VR exposure therapy application for combat-related PTSD. Stud. Health Technol. Inform..

[44] National Research Council (2014). The Context of Military Environments: An Agenda for Basic Research on Social and Organizational Factors Relevant to Small Units.

[45] Mell, J., Lucas, G. M. & Gratch, J. Varied magnitude favor exchange in human-agent negotiation. In *Proceedings of the 20th International Conference on Intelligent Virtual Agents*, vol. 40, 1–8 (2020).

[46] Peled, N., Gal, Y. A. K. & Kraus, S. A study of computational and human strategies in revelation games. In *Proceedings of the 10th International Conference on Autonomous Agents and Multiagent Systems*, 345–352 (2011).

[47] Robu, V., Somefun, D. J. A. & La Poutré, J. A. Modeling complex multi-issue negotiations using utility graphs. In *Proceedings of the 4th International Conference on Autonomous Agents and Multiagent Systems*, 280–287 (2005).

[48] Fatima, S. S., Wooldridge, M. & Jennings, N. R. Approximate and online multi-issue negotiation. In *Proceedings of the 6th international Conference on Autonomous Agents and Multiagent Systems*, 156–163 (2007).

[49] Reeves B, Nass C (1996). The Media Equation: How People Treat Computers, Television, and New Media Like Real People and Places.

[50] Brave S, Nass C (2003). Emotion in Human–Computer Interaction.

[51] Baylor AL (2007). Pedagogical agents as a social interface. Educ. Technol..

[52] Kim Y, Baylor AL (2007). Pedagogical agents as social models to influence learner attitudes. Educ. Technol..

[53] Lee, E. J., Nass, C. & Brave, S. Can computer-generated speech have gender?: An experimental test of gender stereotype. In *Proceedings of CHI Extended Abstracts on Human Factors in Computing Systems*, 289–290 (2000).

[54] de Melo, C. M., Carnevale, P. J. & Gratch, J. Using virtual confederates to research intergroup bias and conflict. In *Proceedings of the Academy of Management*, 11226 (2014).

[55] Dehghani M, Khooshabeh P, Nazarian A, Gratch J (2015). The subtlety of sound: Accent as a marker for culture. J. Lang. Soc. Psychol..

[56] Lee JE, Nass C, Brave S, Morishima Y, Nakajima H, Yamada R (2007). The case for caring colearners: The effects of a computer-mediated colearner agent on trust and learning. J. Commun..

[57] Nass C, Moon Y, Green N (1997). Are machines gender-neutral? Gender-stereotypic responses to computers with voices. J. Appl. Soc. Psychol..

[58] Gilani, S., Sheetz, K., Lucas, G. M. & Traum, D. What kind of stories should a virtual human swap? In *Proceedings of the 16th International Conference on Intelligent Virtual Agents*, 124–136 (2016).

[59] Nowak KL, Biocca F (2003). The effect of the agency and anthropomorphism on users’ sense of telepresence, copresence, and social presence in virtual environments. Presence Teleoper. Virtual Environ..

[60] Bailenson JN, Blascovich J (2004). Avatars.

