# Supplementary material for: Customizing virtual interpersonal skills training applications may not improve trainee performance
Source: Sci Rep. 2023 Jan 3;13:78. doi: 10.1038/s41598-022-27154-2 (PMC9810699; doi:10.1038/s41598-022-27154-2)
Supplement: Supplementary file 1 — Supplementary Information. [file 41598_2022_27154_MOESM1_ESM.docx]

**Appendix 1**

1. Total points earned represents the points the user gains in the negotiation based on the number of items and how much each item is worth. Participants who failed to reach agreement with the agent only received 16 points, which is their BATNA as described above, and these 16 points would be reflected in this measure (accordingly they were removed from the analysis of this variable).

2. Time spent in the negotiation represents the number of seconds users negotiated with the agent. As described above, they had seven minutes in which to negotiate (indicated by the countdown timer), and the amount of time out of this maximum that they utilized (in seconds) indicates greater engagement or further discussion with the agent during the negotiation.

3. Number of offers made is the count of the number of times the user selected an offer on the game-board and hit “send offer” to convey it to the agent. Both full and partial offers count towards the number of offers made by the user.

4. Number of times neutral emoji used is the count of the number of times the user hit the neutral emoji button to convey a neutral expression to the agent. The neutral emoji symbol was then dis-played in the chat field accordingly.

5. Number of times happy emoji used indicates the number of times the user hit the happy emoji but-ton to convey a happy emotional expression. Likewise, the happy emoji symbol was then displayed in the chat field.

6. Number of times angry emoji used tallies the number of times the user hit the angry emoji button to convey an angry expression to the agent, which was displayed in the chat field accordingly.

7. Number of times surprised emoji used is the count of how many times the user hit the surprised emoji button to convey that expression and display it in the chat field.

8. Number of times sad emoji used indicates how many times the user hit the sad emoji button to convey that expression and display it in the chat field.

9. Total number of utterances made represents the superset of all the message statements listed be-low (#11 on). This is a count of all statements, which does not include emojis or offers listed above.

10. Total number of utterances referencing preferences represents the superset of the next two measures listed below, which both include preferences (#11 and #12). These are statements about user’s preferences and questions the user asks about the agent’s preferences.

11. Number of statements made about preferences is the count of all statements the user makes about their preferences. Examples include “I like paintings” or “I like lamps more than records”.

12. Number of questions asked about preferences is the count of all questions the user asks about the agent’s specific preferences. Examples include “Do you like paintings?” or “Do you like lamps more than records?”

13. Number of requests for information is the number of times the user selects a statement that generally asks the agent for information. An example is “So could you tell me about what you want?”

14. Number of false statements about preferences is a subset of statements user makes about their preferences that are not true. Users are told their preferences (as described above) and if they misrepresent their preferences when making a preference statement, it is counted as a false statement about preferences (or a “preferences lie”). For example, one might state that they like records more than lamps (even though lamps are more valuable) in order to make it seem like taking the lamps is a hardship (even though it's what they really want).

15. Number of false statements about BATNA is the number of times user chose to make a statement about their BATNA that was untrue. The users had a slider where they could select their BATNA to represent to the agent (on a scale from 1 to 50). If users selected 16, they would not be strategically misrepresenting the number of points they could get if they walked away from the negotiation (thus would count towards #16 below). However, each time the user chose to represent their BATNA as higher than it actually was (i.e., having a better alternative), it counted as a “BATNA lie.”

16. Number of true statements made about own BATNA, as just described, is the number of times users selected their actual BATNA when indicating to the agent the number of points they could get if they walked away from the negotiation.

17. Number of questions asked about agent’s BATNA, conversely, is the number of times the user asks the agent about its BATNA. An example is “What could you get if you walked away from the negotiation right now?”

18. Number of threats made that are phrased negatively is the number of times the user threatens to walk away, but does it impolitely. An example is “You’re making me want to walk away from this!”.

19. Number of threats made that are phrased positively is the number of times the user threatens to walk away, but does it somewhat politely. An example is “I’m sorry but I think I may walk away.”

20. Number of statements indicating confusion represents the number of times the user selects a statement that suggests what the agent is saying does not make sense to them. An example is “I don’t think that makes sense with previous statements.”

21. Number of times user requests a favor is the number of times that the user selects statements that requests a favor. An example is "Would you please send a good deal in exchange for a favor?"

22. Number of times user says they’re doing them a favor is the number of times that the user selects statements that suggest they are doing the agent a favor. An example is "I'm returning the favor to you! Give me a deal good for you.”

23. Number of generic negative statements made is the number of times a generic negative statement is selected, generally reflecting current dissatisfaction with how the negotiation is going. An example is "I'm not happy with this..."

24. Number of generic positive statements made is the number of times a generic positive statement is selected, generally reflecting current satisfaction with the negotiation. An example is “I'm happy with this so far!”

25. Number of times user accepts an offer is the count of times that the user hits the “accept offer” but-ton to accept the agent’s partial offer (plus the final offer, if one is accepted). Accordingly, the user is making the statement “Yes, I accept that deal,” whether it is a partial offer or the final, full offer.

26. Number of times user rejects an offer, conversely, is the count of times that the user hits the “reject offer” button to reject the agent’s partial or full offer. Accordingly, the user is making the statement “No, I do not accept that deal.”

27. Number of times user requests an offer phrased negatively is the number of times that the user selects statements that request an offer, but does so in a negative way. An example is “What’s wrong with you? Hurry up and make an offer!”

28. Number of times user requests an offer phrased positively is the number of times that the user selects statements that requests an offer, but does so in a positive way. An example is “Would you please make an offer?”
